# O.1.2-7 Optimization of older adults home spaces

**DOI:** 10.1093/eurpub/ckad133.094

**Published:** 2023-09-11

**Authors:** Naureen Akber Ali, Joanne Hudson, Gareth Stratton, Jane Mullins

**Affiliations:** Swansea University, UK; 2132644@swansea.ac.uk; Swansea University, UK; 2132644@swansea.ac.uk; Swansea University, UK; 2132644@swansea.ac.uk; Swansea University, UK; 2132644@swansea.ac.uk

## Abstract

**Purpose:**

There is a lack of studies focused explicitly on the impact of the home physical environment on older adults’ sedentary behaviour (SB) and physical activity (PA). Therefore, the present study aims to investigate older adults’ perception of their home environment and its impact on their PA and SB.

**Methods:**

A qualitative exploratory research design, based on in-depth interviews (IDIs) and focus groups discussions (FGDs) with older adults was conducted from 1st August 2022 to 31st December 2022. We identified four major themes: (I) Home layout and PA, (II) Space designation within the home, (III) Electronic equipment, furniture and material within the home space, (IV) Changing infrastructure within the home space. We analysed the data through reflexive thematic analysis using NVivo 10 software.

**Results:**

Older adults valued the physical environment, and they managed their indoor space to control environment by structuring and restructuring to make it conducive for PA. Outdoor space such as garden was also an important factor for older adults’ PA. Moreover, older adults being autonomous and without any restrictions spent a substantial amount of time on utilizing electronic media. Further modification (such as adaptive aids, modification in the bathroom and etc) within the home space assist them in daily activities.

**Conclusion:**

The findings suggest that both indoor and outdoor space play an important role in enhancing PA level and minimizing SB. Although space is important but how space is used, and its intention of use is also very crucial. The study finding also supported and recommended the notion of ‘think small for large effects’ within the home space. Instead of building the indoor facilities (such as indoor gyms, swimming pool and etc) older adults should mapped out small-scale activities such as avoiding screens in bedroom or removing clutter to increase space for activity.

